# Distinct Timing Mechanisms Produce Discrete and Continuous Movements

**DOI:** 10.1371/journal.pcbi.1000061

**Published:** 2008-04-25

**Authors:** Raoul Huys, Breanna E. Studenka, Nicole L. Rheaume, Howard N. Zelaznik, Viktor K. Jirsa

**Affiliations:** 1 Theoretical Neuroscience Group, UMR 6152 Institut des Sciences du Mouvement, CNRS and Université de la Méditerranée, Marseille, France; 2 Purdue University, Health and Kinesiology, West Lafayette, Indiana, United States of America; 3 Center for Complex Systems and Brain Sciences, Physics Department, Florida Atlantic University, Boca Raton, Florida, United States of America; University College London, United Kingdom

## Abstract

The differentiation of discrete and continuous movement is one of the pillars of motor behavior classification. Discrete movements have a definite beginning and end, whereas continuous movements do not have such discriminable end points. In the past decade there has been vigorous debate whether this classification implies different control processes. This debate up until the present has been empirically based. Here, we present an unambiguous non-empirical classification based on theorems in dynamical system theory that sets discrete and continuous movements apart. Through computational simulations of representative modes of each class and topological analysis of the flow in state space, we show that distinct control mechanisms underwrite discrete and fast rhythmic movements. In particular, we demonstrate that discrete movements require a time keeper while fast rhythmic movements do not. We validate our computational findings experimentally using a behavioral paradigm in which human participants performed finger flexion-extension movements at various movement paces and under different instructions. Our results demonstrate that the human motor system employs different timing control mechanisms (presumably via differential recruitment of neural subsystems) to accomplish varying behavioral functions such as speed constraints.

## Author Summary

A fundamental question in motor control research is whether distinct movement classes exist. Candidate classes are discrete and continuous movement. Discrete movements have a definite beginning and end, whereas continuous movements do not have such discriminable end points. In the past decade there has been vigorous, predominantly empirically based debate whether this classification implies different control processes. We present a non-empirical classification based on mathematical theorems that unambiguously sets discrete and continuous rhythmic movements apart through their topological structure in phase space. By computational simulations of representative modes of each class we show that discrete movements can only be executed repetitively at paces lower than approximately 2.0 Hz. In addition, we performed an experiment in which human participants performed finger flexion-extension movements at various movement paces and under different instructions. Through a topological analysis of the flow in state space, we show that distinct control mechanisms underwrite human discrete and fast rhythmic movements: discrete movements require a time keeper, while fast rhythmic movements do not. Our results demonstrate that the human motor system employs different timing control mechanisms (presumably via differential recruitment of neural subsystems) to accomplish varying behavioral functions such as speed constraints.

## Introduction

Discrete movements constitute singularly occurring events preceded and followed by a period without motion (i.e., with zero velocity) for a reasonable amount of time, such as a single finger flexion or flexion-extension cycle [1],[2]. Continuous movements lack such recognizable endpoints, and normally are considered rhythmic if they constitute repetitions of particular events, in which case they often look quite sinusoidal. While it is trivial that discrete movements can be repeated periodically, the question whether motor behavior is fundamentally discrete or rhythmic is not. Is motor behavior fundamentally discrete, reducing rhythmic movement to mere concatenations of discrete movements [3],[4]? Or is motor control fundamentally rhythmic, in which case discrete movements are merely ‘aborted’ cycles of rhythmic movements [5]–[7]? Alternatively, both types of movements may belong to distinct classes that are irreducible to each other [8]–[10], hence implying the utilization of different movement generating mechanisms.

Proponents of the ‘discrete perspective’ have sought evidence for discrete movement control through the identification of movement segments in movement trajectories. However, segmented motion need not imply segmented control [11]. In fact, the possibility to settle the dispute (solely) on the basis of kinematic features of movement (movement time, peak velocity, symmetry of velocity profiles, etc.) has recently been questioned [12]. Other researchers have aimed to identify the neural structures associated with discrete and rhythmic movements. For instance, Schaal and colleagues [9] showed that the brain areas that were associated with rhythmic movements were approximately a subset of those that were active during discrete movement execution. Differential involvement of neural subsystems does not provide a classification principle, however. Unambiguous classification requires the identification of invariance that is unique to each class so that the intersection of these two sets of characteristics is empty. Such a result will provide unambiguous evidence that two classes indeed are distinct. Dynamic systems theory offers such a classification principle based on phase flow topologies, which identify all behavioral possibilities within a class. Its significance lies in the fact that the classification is model-independent; every behavior within a class can be mapped upon others, whereas maps between classes do not exist. We use this principled approach to address the controversy whether discrete and rhythmic movements are fundamentally different. To that aim, we introduce the notion of phase flow topologies, identify the invariance separating two movement classes, and present an experimental study testifying to the existence of (at least) two different movement classes.

**Figure 1:**
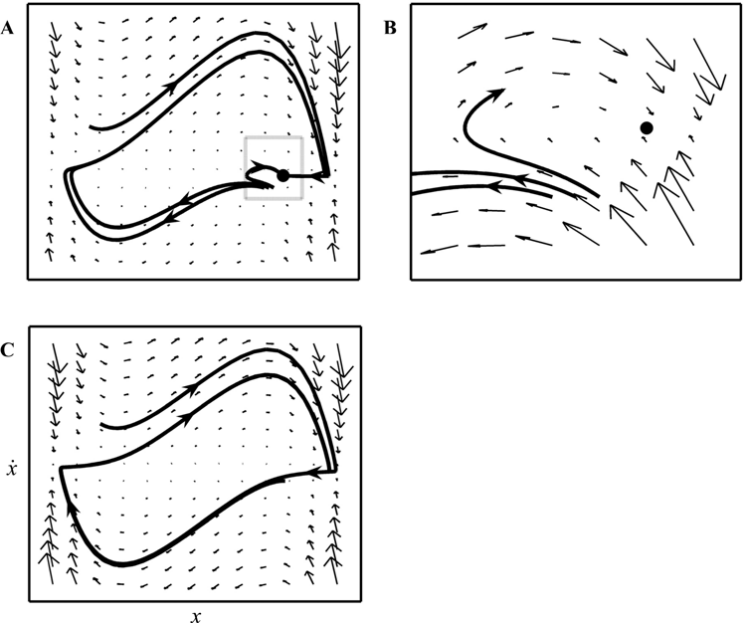
Phase space topologies. The small arrows delineate the phase flow. Horizontal axes represent position (*x*); vertical axes represent velocity (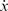) (only indicated in [C]). (A–B) Stable fixed point and separatrix. A close-up of the dotted-boxed area in (A) is provided in (B). A stable fixed point is represented by the black point; arrows converge to it. The divergence of nearby starting trajectories reveals locally distinct flows set apart by a separatrix. (C) Stable limit cycle.

Deterministic, time-continuous and autonomous systems can be unambiguously described through their flow in state (or phase) space, defined as the space spanned by the system's position *x* and velocity 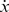 (under the commonly adopted assumption that the deterministic component of movement trajectories can be fully described by two state variables). Whereas the phase flow quantitatively describes the system's evolution as a function of its current state (*x*, 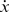); the system's qualitative behavior is solely determined by its phase flow topology. From the Poincaré-Bendixson theorem [13],[14] it follows that the only possible topologies in two dimensional systems are composed of elements referred to as fixed points, limit cycles, and separatrices. A fixed point of the system identifies a rest state (i.e., rate of change is zero, 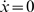), and, if stable, all trajectories in phase space eventually converge to it (Figure 1A). A system located at a fixed point can only depart from it in the presence of an external stimulation. A separatrix is a subset of points in the phase space that divides locally distinct phase flows (Figure 1A and 1B). In most cases for two-dimensional phase spaces, a separatrix is a line from which the flow points away in approximately opposite directions. Even simpler, for one-dimensional phase spaces any unstable fixed point is a separatrix. Limit cycles (Figure 1C) are closed loops in a two-dimensional phase space. If a limit cycle is stable, then all trajectories converge to it. A system on a limit cycle will repetitively traverse the same trajectory in phase space and sustain a periodic motion. Since these elements, fixed points and limit cycles, compose all phase flows in two dimensions, we associate discrete and rhythmic movements with these. The Hartman-Grobman theorem [13],[14] states that the flow in the local neighborhood of a fixed point is topologically equivalent to that of its linearization, which implies that a continuous invertible mapping (a homeomorphism) between both local phase spaces exists. From these theorems it follows that dynamical systems belong to the same class if, and only if, they are topologically equivalent. Therefore, movements that can be shown to be governed by fixed point dynamics versus movements governed by limit cycle dynamics are not reducible to each other, and as such we can make the strong claim that they are from different equivalence classes.

In consideration of the notion of topological equivalence, Jirsa and Kelso [15] recently formulated a generic model construct that allows for a stable fixed point and a separatrix (referred to as the mono-stable regime) or a stable limit cycle regime (Figure 1) in its corresponding phase space (see Text S1). These topologies correspond to single (i.e., discrete) flexion-extension movements and rhythmic movement, respectively. This perspective has three crucial features. First, the qualitative behavior in each regime is model independent. Second, each single movement execution in the mono-stable regime depends on an external triggering (mathematically speaking, the system is non-autonomous). In contrast, in the (autonomous) limit cycle regime no external stimulation is required and movement is self-sustaining. Third, the phase flow underlying movement is invariant on the time scale of the movement in both cases. Here, we examine this perspective by directly investigating numerically generated phase flows as well as those generated by humans and show that discrete and continuous movements belong to distinct dynamical classes.

## Results

**Figure 2:**
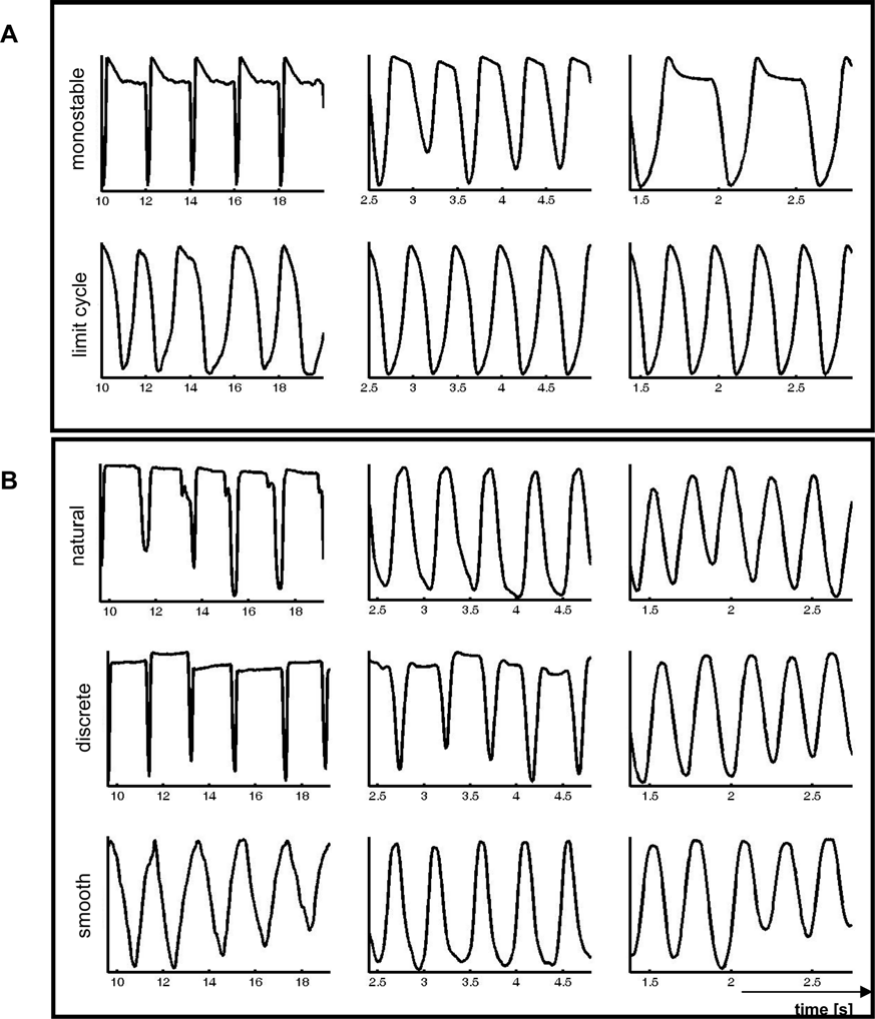
Representative time-series. Time [s] is represented on the horizontal axes; (normalized) position on the vertical axes (not depicted in the Figure). (A) Model simulations in the mono-stable (upper panel) and limit cycle regime (lower panel) at 0.5 Hz, 2.0 Hz, and 3.5 Hz (left, middle, and right column, respectively). (B) Data of one participant in the natural, discrete, and smooth condition (upper, middle, and lower row, respectively) at 0.5 Hz, 2.0 Hz, and 3.5 Hz (left, middle, and right column, respectively). Note the qualitative correspondence with the mono-stable regime in the discrete and natural condition at slow movement paces and with the limit cycle regime in all conditions at high paces.

We computationally examined the generic model under a large parameter and frequency range in order to examine the robustness and limits of its behavior in both dynamical regimes (see Materials and Methods). In the limit cycle regime, the timing requirement (i.e., the computationally implemented movement frequency) was met under all movement paces (i.e., frequencies). In contrast, in the mono-stable regime the actual timing deviated from the required timing due to a period-doubling when the movement pace exceeded approximately 2.0 Hz. (Figure 2A), which occurs due to the arrival of stimulus *n* before movement *n*−1 has finished. These observations were robust under all parameter settings within each dynamical regime (see Text S1 and Figures S1, S2, and S3), although the frequency at which the period doubling occurred showed a small variation as a function of one of the model parameters. In fact, while the exact frequency at which stimulus – movement interference occurs will show little variation as a function of the specific model realization (i.e., through function *g_1_* and *g_2_*; see Equation 1 in Text S1), its occurrence with increasing frequency of stimulation is unavoidable. By implication, every discrete movement system has an upper (frequency) limit in generating sequential movements.

In the behavioral experiment human participants (*n* = 8) performed an auditory-paced unimanual finger flexion-extension timing task under similar movement paces (from 0.5 Hz to 3.5 Hz; step size 0.5 Hz) that were presented in ascending or descending order (see Materials and Methods). The participants were instructed to synchronize their full flexion with the metronome under three instruction conditions: to move as fast as possible (with staccato like movements being initiated to end/start a cycle), as smooth as possible (move so that the finger is continuously moving during the movement period interval) or without any specific instruction. We refer to these conditions as ‘discrete’, ‘smooth’, and ‘natural’, respectively (Figure 2B). Please note that, notwithstanding the repetitiveness of the movements, these instructions may elicit movements generated by distinct control mechanisms but do not prescribe the latter.

**Figure 3:**
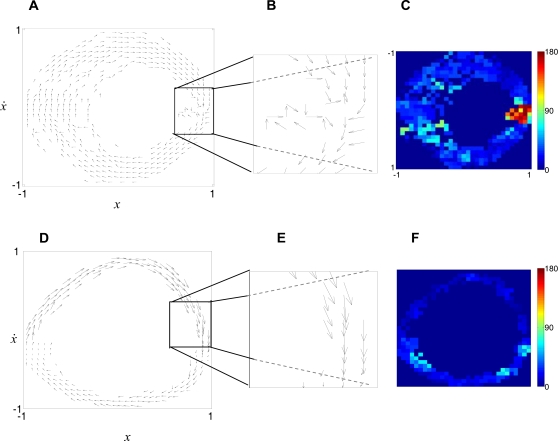
Reconstructed vector field and corresponding angles between neighbouring phase flow vectors corresponding to five trials from one participant. Horizontal axes represent normalized position (*x*); vertical axes represent normalized velocity (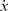) (only indicated in [A] and [D]). (A) Reconstructed vector field for the discrete condition at 0.5 Hz (left; see text and Data Analysis). (B) Enlarged representation of the boxed area in (B). (C) Corresponding angle diagram. While the existence of a fixed point (vectors with different directions pointing towards a point [i.e., the arrowheads converge]) and a separatrix (that locally divides the space in distinct flows; vectors with different directions pointing away from a point [i.e., the arrowheads diverge]) can be directly glanced from (B), they have to be inferred from (C). The existence of locally opposing angles, however, necessarily implies the presence of a fixed point and a separatrix. (D–F) Equivalent representations as in (A–C) corresponding to five trials from one participant in the ‘discrete’ condition at 3.5 Hz. Vectors inside and outside the limit cycle point slightly towards it while being close to parallel to it.

**Figure 4:**
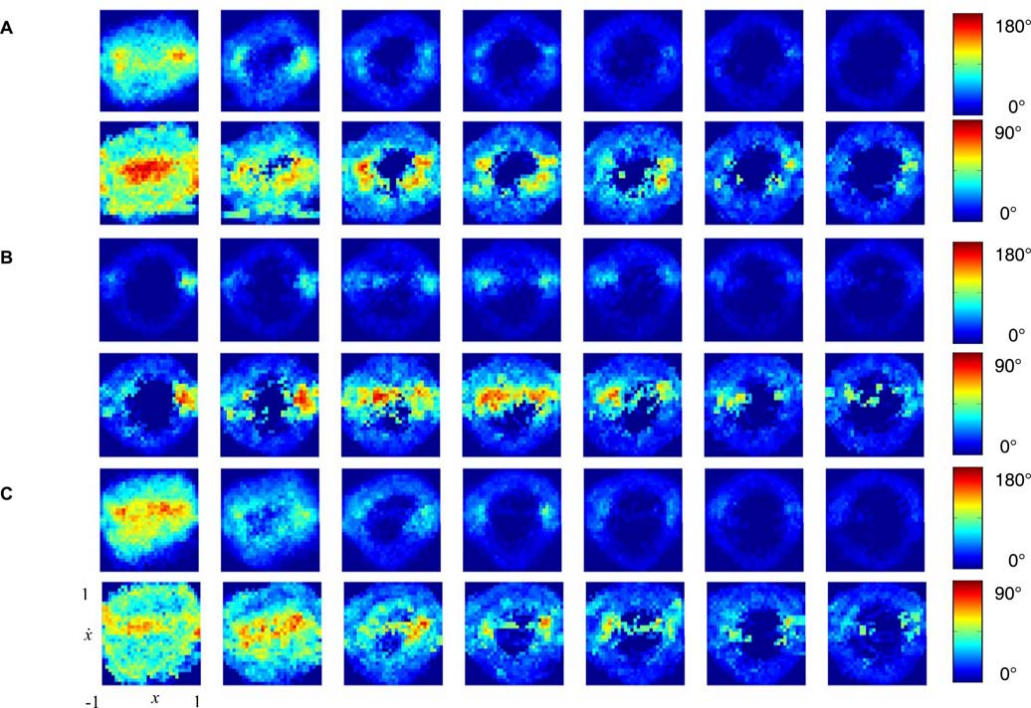
Reconstructed angle diagrams averaged across all participants as a function of movement pace and instruction condition. Horizontal axes represent normalized position (*x*); vertical axes represent normalized velocity (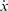) (only indicated in lower left panel). (A) For the natural condition. (B) For the discrete condition. (C) For the smooth condition. For (A–C) the mean and standard deviation are depicted in the upper and lower row, respectively. The magnitude of the angles is represented through colour coding (right side of each panel).

We reconstruct the vector fields underlying the phase flow (see Figure 3 and Materials and Methods) using a novel technique [16],[17] that has been successfully tested on simulated data from dynamical systems [18],[19] and applied in fields like (among others) physics [16],[17], engineering [20], economics [21], and which was recently introduced in the study of human movement [19],[22],[23]. In addition, we investigate the phase spaces in terms of two-dimensional probability distributions and performed more ‘traditional’ kinematic analysis commonly utilized in the (human) movement sciences (see Text S1 and Figures S4, S5, S6, S7, S8, and S9). Figure 3 represents the vector fields (Figure 3A, 3B, 3D, 3E) from five trials of a single participant and the corresponding angle diagrams (Figure 3C and 3F, respectively), and clearly indicates the existence of a fixed point (Figure 3A–3C) and a limit cycle (Figure 3D–3E). Figure 4A–4C (upper row for each subfigure) shows the angle diagrams averaged across all participants for each frequency and instruction condition. Obviously, the averaging across participants, to some extent, smears out the representation of the topological structures, as indicated by the standard deviations across participants of the angle reconstructions in the lower rows of Figure 4A to 4C. Regardless, the existence of a single fixed point at slow movement paces in the discrete condition, indicating the utilization of the mono-stable regime dynamics, can be appreciated from Figure 4A (upper row). In the natural and smooth condition the vector fields are less structured at slow paces, especially at 0.5 Hz (Figure 4A–4C). Scattered (to some degree) vector fields and the existence of either one or two fixed points appear at 0.5 Hz in the smooth and the natural condition. The fixed point(s) appears clearer at 1.0 Hz to 2.0 Hz in both conditions. Under all instruction conditions, however, the fixed point(s) vanishes at high movement paces and invariantly gives way to limit cycle dynamics (Figure 4A–4C). These results indicate that humans utilize distinct timing mechanisms in a movement pace-dependent manner.

## Discussion

What are the implications of these finding? First and foremost, our results lay the foundation of a motor behavior classification scheme based on mathematical theorems. We demonstrated that discrete and fast rhythmic movements constitute distinct classes; their genesis is, by implication, underwritten by different mechanisms. Fast rhythmic movements are autonomous and their timing emerges from the movement dynamics. In contrast, discrete movements are non-autonomous: Their timed execution cannot originate from their dynamics and hence requires external time keeping, most likely arising from a neural structure or network that is not implicated in the implementation of the dynamics. In that regard, the discrete movements studied here constituted full, repetitive (flexion-extension) cycles. Similar movements are sometimes referred to as continuous movements in the presence of temporal events [24],[25]. We refer to them as ‘discrete’ as they are governed by fixed point dynamics. Regardless, please note that even though in many cases the exact timing of a discrete movement is hardly of importance, every discrete movement initiation (be it embedded in a regular or irregular sequence of movements or not) requires ‘external’ stimulation, which is ultimately timed. This also holds for an additional class of discrete movements, namely, point-to-point movements (cf. [9]), in which two stable fixed points exist simultaneously (see Supporting Information, and [15]). While our findings are by and large in line with the more ‘traditional’ and purely behaviorally-defined classification [2] as well as recent versions thereof in terms of movement continuity [24],[25], they also identify their limitation; continuous movements do not constitute a single class. This limitation indeed strengthens our call for a classification of movement rooted in mathematical theory that bears directly on the mechanisms underlying movement genesis.

The movements at a slow pace, in particular at 0.5 Hz, under the ‘smooth’ instruction (and for some participants under the ‘natural’ instruction) were invariantly characterized by (relatively) irregular phase flows (see Figure 4C). The Poincaré-Bendixson theorem [13],[14] rules out topological structures other than fixed points (and separatrices) and limit cycles in two-dimensional phase space. The (relatively) irregular phase flows (with indices of multiple fixed points) may (by hypothesis) represent movements whose phase flow changes on a similar time scale as the movement. Such flows can be predicted for equilibrium point models [4]–[6] that, from a dynamical perspective, can be interpreted in terms of (the relocation of) a fixed point [26]. In fact, phase flow changes on the time scale of the movement also underwrite an alternative dynamical model [7]. Accordingly, discrete movements are accounted for by the destabilization and subsequent stabilization of fixed points interspersed by a time interval in which a limit cycle exists that effectively generates the (discrete) movement. The destabilization is accounted for by an external impact relative to the dynamics (‘behavioral information’). In other words, discrete movement generation is non-autonomous according to this account also.

The notion of time keepers versus timing resulting from movement dynamics are not new. On the contrary, these notions are central to two distinct theoretical camps (the information processing perspective and dynamical system approach, respectively) that have little interaction ([27]; and see e.g., the special issue of *Brain & Cognition 48*, 2002). The notion of a time keeper (or central timer) became firmly established by the well-known two-level timing model [28],[29]. Accordingly, the behavioral expression in tapping movements – the often observed negative correlation between consecutive tapping intervals – is the resultant of the repetitive movement initiation by a central time keeper and the impact of the motor delays preceding and following each particular tap (which are all random variables). Notwithstanding the various elaborations of (‘cognitive’) timing models ever since [30]–[33], the notion of time keeping is inherently connected with abstract mental representations. In contrast, eschewing representational concepts, dynamicists view timing and coordination as properties arising from (self-organized) pattern formation processes [34]–[37]. Here, we elaborated on two distinct dynamical organizations and report evidence that humans ‘implement’ either of these depending on movement rate. In the non-autonomous scenario movement initiation (and thus timing) depends on a mechanism external to the dynamics. While we framed this in terms of time keeping, this should not be taken to imply that we adhere to a representational account thereof (cf. [36]). In other words, the non-autonomous case should not be simply equated with a dynamical version of a two-level model (notwithstanding the – to some extent superficial – similarity in terms of a distinction between ‘clock’ and ‘motor’ components).

The implication of external timekeeper during discrete movements begs the question what neural structure(s) could fulfill this function? Spencer and colleagues [25] showed that patients with cerebellar lesions have deficits in producing discontinuous but not continuous movements, which supports the idea that the cerebellum is implicated in timing in the non-autonomous but not autonomous case (see also [38]–[40]). However, Schaal and colleagues [9], using fMRI, reported contralateral activity in several non-primary motor areas and the cerebellum during discrete wrist movements that was absent during their rhythmic counterparts. This result favors the suggestion that timing is a property originating from a distributed neural network [41],[42]. Indeed, the neural basis underlying timing remains yet to be elucidated. Implementing the present paradigm in the context of brain imaging may help establishing that aim.

Finally, it has been repeatedly suggested that motor control is simplified through the use of ‘motor primitives’, the motor system's elements thought of as its ‘building blocks’. The modular organization of the vertebrae spinal motor system and the reproducibility of specifically coordinated muscle activity upon stimulation of certain modules (neural circuits) instigated the idea that motor behavior is organized along such hard-wired structures [43]–[45]. On a more abstract level, the two timing architectures we identified here qualify as candidate building blocks in human motor control.

## Materials and Methods

### Computational Analysis

We numerically investigate the equation
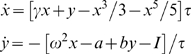
in which *a* and *b*, and *γ*, represent parameters, *ω* represents the system's eigenfrequency, *τ* represent a time constant, and *I* the external stimulation. For all simulations we use *τ* = 1, and if applicable, a stimulus duration corresponding to 80 ms and magnitude of 3.5.

For the mono-stable regime, the following parameter settings are implemented: *γ* = 1; *ω* = 1; *a* = [1.01, 1.09] with steps of 0.02; *b* = [−0.1, 0.8] with steps of 0.1; and *I* = [0.25 Hz, 4.00 Hz] with steps of 0.25 Hz. For the limit cycle regime, the implemented parameters are: *a* = 0; *b* = [−0.2, 0.3] with steps of 0.1; and *ω* = [0.25 Hz, 4.00 Hz] with steps of 0.25 Hz. For each frequency *ω*, *γ* is chosen to as to ensure that the system oscillates with the appropriate frequency. All simulations are performed using a fourth-order Runge-Kutta method. Gaussian white noise ξ(*t*) is added to the evolution equations of the *y*-variable, where 〈ξ(*t*)〉 = 0, 〈ξ(*t*)ξ(*t*)〉 = *Q* ^2^δ(*t*−τ), Q = 0.01. The triangular brackets 〈·〉 denote time averages.

### Participants

Eight participants (mean age = 27.9 years) took part in the experiment. Seven participants were (self-reported) right-handed, one participant was left-handed. Participants reported an average of 2.75 years of musical experience with a minimum of 0 years and a maximum of 8 years. The protocol was approved by the Purdue University Committee on the Usage of Human Research Participants and was in agreement with the Declaration of Helsinki. Informed consent was obtained from all participants.

### Movement Recording

Data were collected using a Polhemus Liberty-8 receiver (23×13×11 mm, 4 gm) that was affixed to the participant's index finger with adhesive tape. This receiver was controlled by Matlab using an AuSIM-AuTrakMatlab USB driver and collection interface via library C++ calls. Three dimensional position data were collected at 240 Hz. The motion in the medio-lateral direction was used for further analysis.

### Task and Procedure

The flexion-extension movements were performed in the transverse plane involving no physical contact with any object. During the performance, the participants were seated at a 77-cm high table, and each participant rested the medial portion of his or her hand on a padded wooden block and Velcro held their hand in place. Ten trials were performed under three instruction conditions. Under each instruction condition, the participant was instructed to time the full finger flexion with the occurrence of the metronome tone. The instruction for the ‘natural’ condition was to do so in a manner that felt most natural. The instruction for the ‘smooth’ condition was to execute the movements as smooth (sinusoidal) as possible so as to be moving always ‘at an even pace’. For the ‘discrete’ condition the instruction was to execute each complete flexion and extension movement as quickly as possible. In each condition five trials were performed with increasing metronome pace (from 0.5 Hz to 3.5 Hz; step size 0.5 Hz) and five trials with decreasing pace. Every frequency plateau lasted for 15 tones. Participants were instructed to quickly and smoothly adjust to changes in pace. A 30 second rest interval was provided between trials. Feedback was given after a trial if the participant's average cycle duration for any of the seven metronome paces had deviated more than 15 percent of the goal interval duration. The order of increasing or decreasing set of trials was performed in a blocked design. All participants performed the first condition (‘natural’) on day one. The order of the other two conditions was balanced for all participants. Each session lasted approximately one and a half hour.

### Data Analysis

Human movement is inherently stochastic; its dynamics constitutes a deterministic and a stochastic (i.e., random) component [19],[34],[35]. The future state of a stochastic process is conditional upon the probability for its state to be at a given time instant at a specific point in phase space, which can be described by probability distributions [34],[46]. The computation of probability distributions allows one to disentangle the deterministic and stochastic dynamical components underlying stochastic processes [16]–[19]. Here, we extract these components to focus on the deterministic dynamics. Thereto, for each trial, we computed the movement velocity and normalized all position (*x*) and velocity (*y*) time-series to the interval [−1, 1]. Next, using a grid size of 31, we computed for all trials the conditional probability matrix, *P*(*x*,*y*,*t*|*x_0_*,*y_0_*,*t_0_*), that is, the probability to find the systems at state (*x*,*y*) at a time *t* given its state (*x_0_*,*y_0_*) an earlier time step *t_0_*. Subsequently, we computed the Kramers-Moyal coefficients [16]–[20] representing the drift coefficient according to
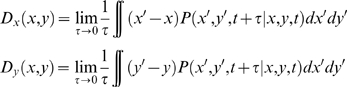



The coefficients *D_x_* and *D_y_* were averaged across the five trial repetitions for each participant, instruction condition and movement frequency. From the first two coefficients (that represent the *x*-, and *y*-component of the corresponding velocity vector), we computed for each bin the angle *θ* between its corresponding velocity vector and that of each of its neighbors (provided their existence) according to
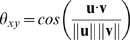
in which **u** and **v** represent two neighboring vectors defined by *D_x_*(*x,y*) and *D_y_*(*x,y*) at position *x* and *y* in phase space. Next, we extracted the maximal value of *θ* in phase space. The existence of locally opposing vectors (i.e., with an angle of approximately 180°) indicate the existence of a fixed point. We then computed for each instruction condition×movement frequency condition the mean and standard deviation of the maximal angle across participants and frequency order.

## Supporting Information

Figure S1 Probability density distributions. The position and velocity axes are indicated in the lower right panel, and the extracted 3-bin summed probability values are provided for each distribution. (A) Probability density distributions of model simulations in the mono-stable regime (upper panel) and limit cycle regime (lower panel) at 0.5 Hz, 2.0 Hz, and 3.5 Hz (left, middle, and right column, respectively). The cycle period always corresponds to the required frequency except for the mono-stable regime at 3.5 Hz, due to a period doubling. (B) Probability density distributions of the data of one participant in the discrete, natural, and smooth condition (upper, middle, and lower row, respectively) at 0.5 Hz, 2.0 Hz, and 3.5 Hz (left, middle, and right column, respectively).
(5.28 MB TIF)

Figure S2 Symmetry ratios in the mono-stable regime. The symmetry ratio of the simulated data in the mono-stable regime is presented as a function of parameter b and frequency.
(0.76 MB TIF)

Figure S3 Spectral power in the mono-sable regime. The amount of spectral power in the mono-stable regime as a function of parameter b and frequency at the sub-harmonic (P[*ω*/2]) (left panel), the fundamental frequency (P[*ω*]) (middle panel), and the first super-harmonic (P[2*ω*]) (right panel).
(0.59 MB TIF)

Figure S4 Symmetry ratios of the human data. The average symmetry ratio for the participants (n  =  8) adopting a ‘discrete’ motor solution (D; n  =  4) and a ‘smooth’ motor solution (S; n  =  4) in the natural condition as a function of frequency for the natural, discrete, and smooth condition (left, middle, and right panel, respectively). The vertical bars indicate standard deviations.
(0.91 MB TIF)

Figure S5 Spectral power in the human data. The amount of spectral power in the human data as a function of instruction condition and frequency at the sub-harmonic (P[*ω*/2]) (left panel), the fundamental frequency (P[*ω*]) (middle panel), and the first super-harmonic (P[2*ω*]) (right panel). For the natural conditions, the data for the participants who adopted the ‘discrete’ and ‘smooth’ condition (Nd and Ns, respectively) are depicted separately, whereas for the discrete and smooth condition thee data are collapsed across both groups.
(0.88 MB TIF)

Figure S6 Goal frequency versus observed frequency. Note that in conditions where participants were slowing down, the observed frequency values are plotted in the reverse order of which they were performed.
(0.24 MB TIF)

Figure S7 Goal frequency versus coefficient of variation. Note that in conditions where participants were slowing down, the CVs are plotted in the reverse order of which they were performed.
(0.27 MB TIF)

Figure S8 Goal frequency versus normalized mean squared jerk. Note that in conditions where participants were slowing down, the values of jerk are plotted in the reverse order of which they were performed.
(0.27 MB TIF)

Figure S9 Goal frequency versus percentage of time to peak negative velocity. Note that in conditions where participants were slowing down, the values of percent time to peak negative velocity are plotted in the reverse order of which they were performed.
(0.29 MB TIF)

Text S1 Supporting information.
(0.07 MB DOC)
