# Supplementary figures and images for: Distinct Timing Mechanisms Produce Discrete and Continuous Movements
Source: PLoS Comput Biol. 2008 Apr 25;4(4):e1000061. doi: 10.1371/journal.pcbi.1000061 (PMC2329590; doi:10.1371/journal.pcbi.1000061)

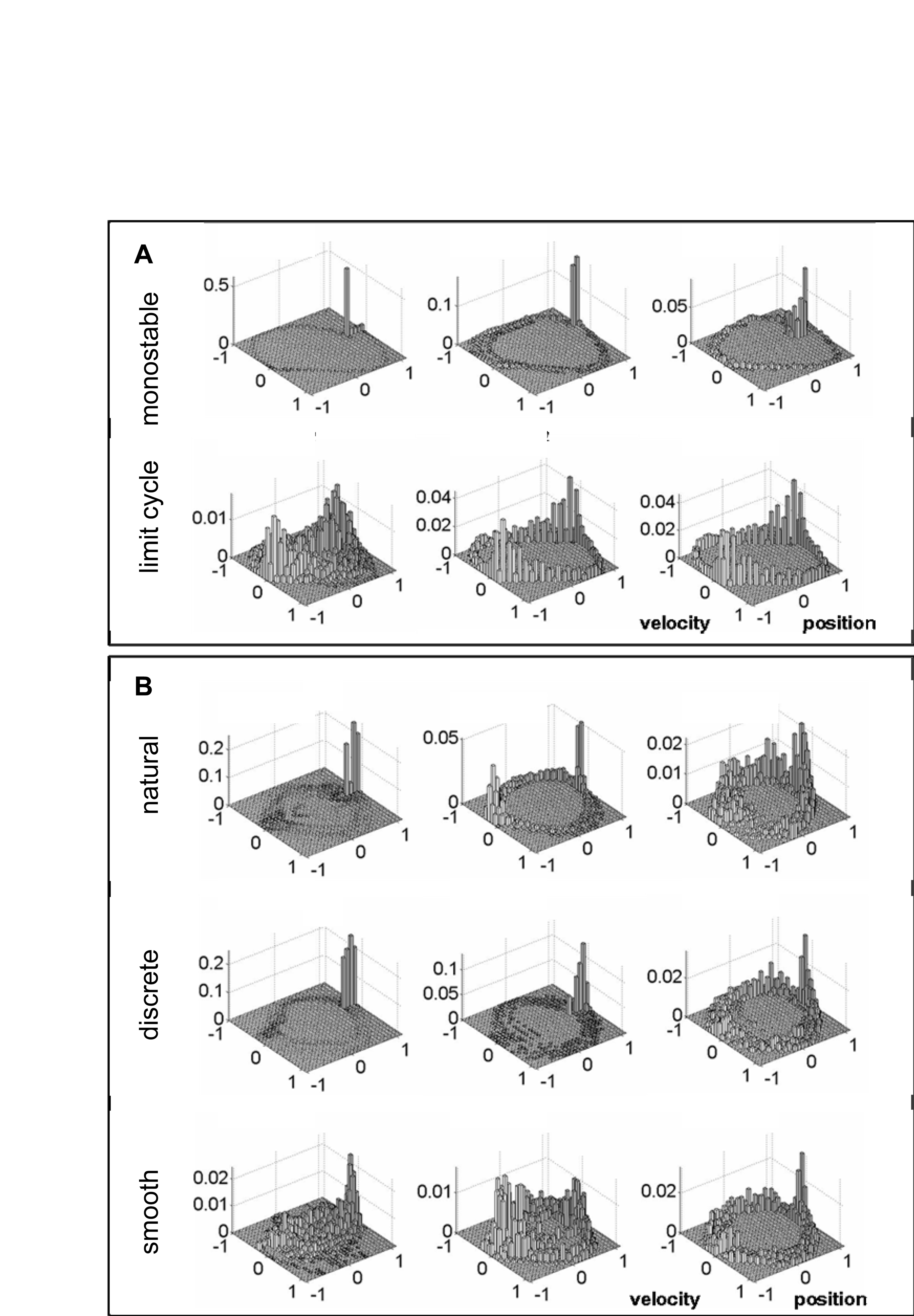

Supplement: Figure S1 — Probability density distributions. The position and velocity axes are indicated in the lower right panel, and the extracted 3-bin summed probability values are provided for each distribution. (A) Probability density distributions of model simulations in the mono-stable regime (upper panel) and limit cycle regime (lower panel) at 0.5 Hz, 2.0 Hz, and 3.5 Hz (left, middle, and right column, respectively). The cycle period always corresponds to the required frequency except for the mono-stable regime at 3.5 Hz, due to a period doubling. (B) Probability density distributions of the data of one participant in the discrete, natural, and smooth condition (upper, middle, and lower row, respectively) at 0.5 Hz, 2.0 Hz, and 3.5 Hz (left, middle, and right column, respectively). (5.28 MB TIF) [file pcbi.1000061.s001.tif]

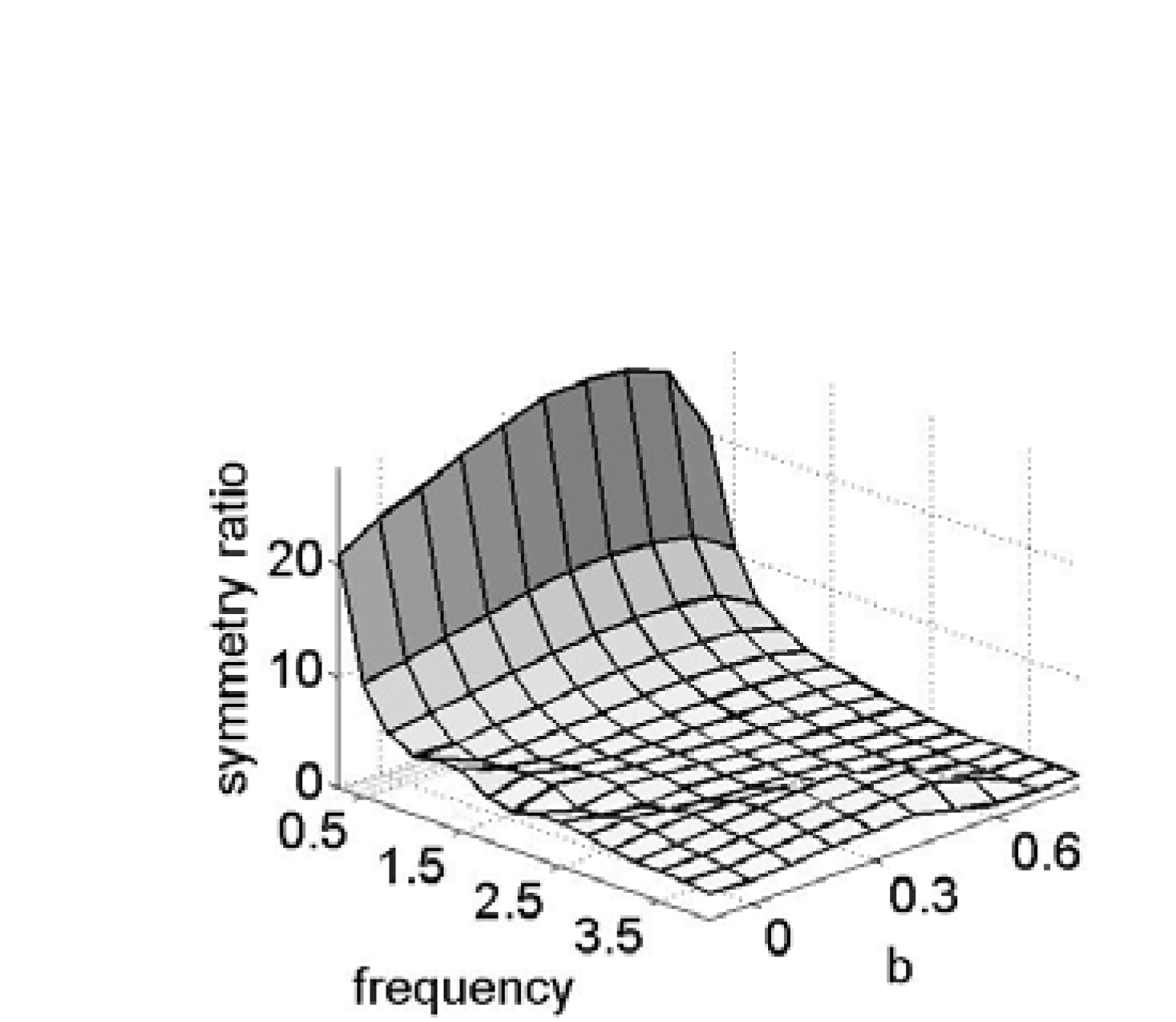

Supplement: Figure S2 — Symmetry ratios in the mono-stable regime. The symmetry ratio of the simulated data in the mono-stable regime is presented as a function of parameter b and frequency. (0.76 MB TIF) [file pcbi.1000061.s002.tif]

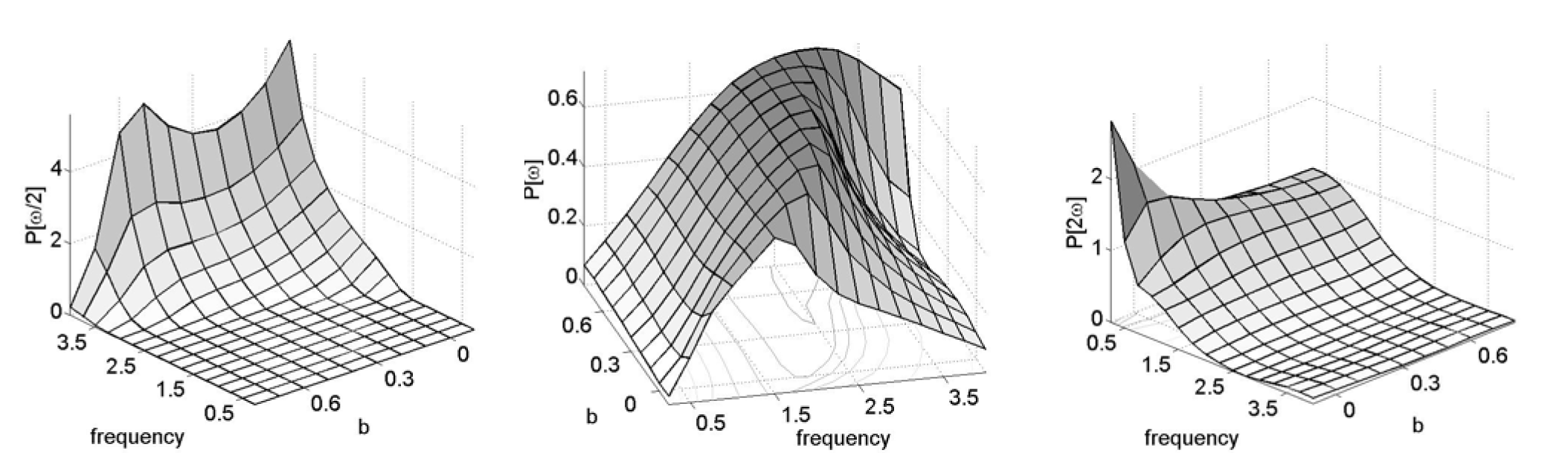

Supplement: Figure S3 — Spectral power in the mono-sable regime. The amount of spectral power in the mono-stable regime as a function of parameter b and frequency at the sub-harmonic (P[ω/2]) (left panel), the fundamental frequency (P[ω]) (middle panel), and the first super-harmonic (P[2ω]) (right panel). (0.59 MB TIF) [file pcbi.1000061.s003.tif]

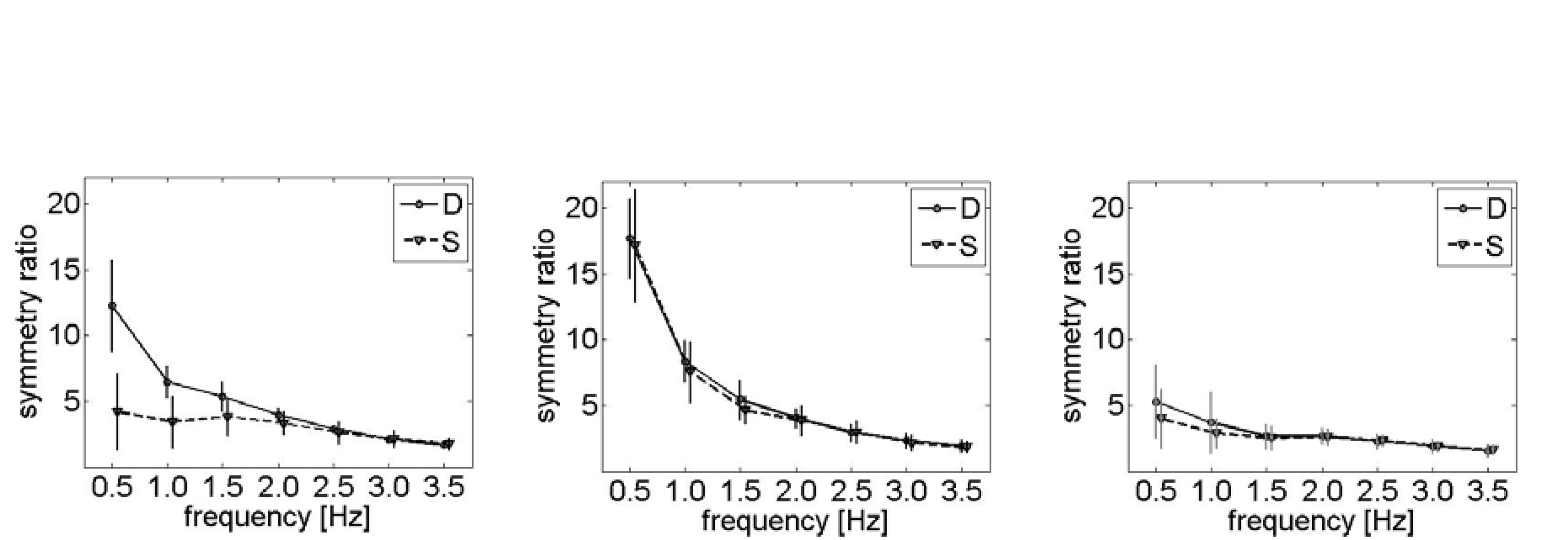

Supplement: Figure S4 — Symmetry ratios of the human data. The average symmetry ratio for the participants (n = 8) adopting a ‘discrete’ motor solution (D; n = 4) and a ‘smooth’ motor solution (S; n = 4) in the natural condition as a function of frequency for the natural, discrete, and smooth condition (left, middle, and right panel, respectively). The vertical bars indicate standard deviations. (0.91 MB TIF) [file pcbi.1000061.s004.tif]

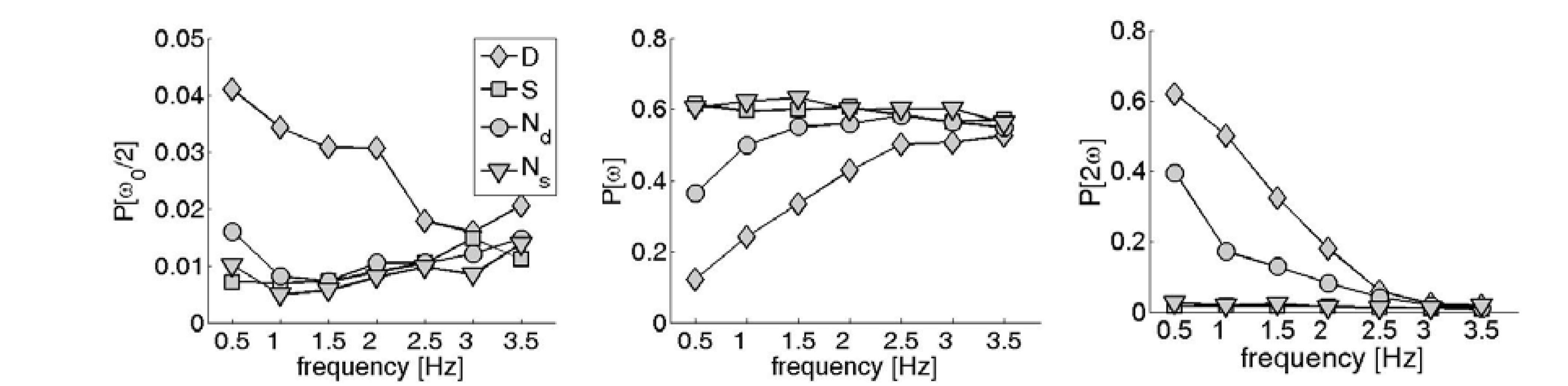

Supplement: Figure S5 — Spectral power in the human data. The amount of spectral power in the human data as a function of instruction condition and frequency at the sub-harmonic (P[ω/2]) (left panel), the fundamental frequency (P[ω]) (middle panel), and the first super-harmonic (P[2ω]) (right panel). For the natural conditions, the data for the participants who adopted the ‘discrete’ and ‘smooth’ condition (Nd and Ns, respectively) are depicted separately, whereas for the discrete and smooth condition thee data are collapsed across both groups. (0.88 MB TIF) [file pcbi.1000061.s005.tif]

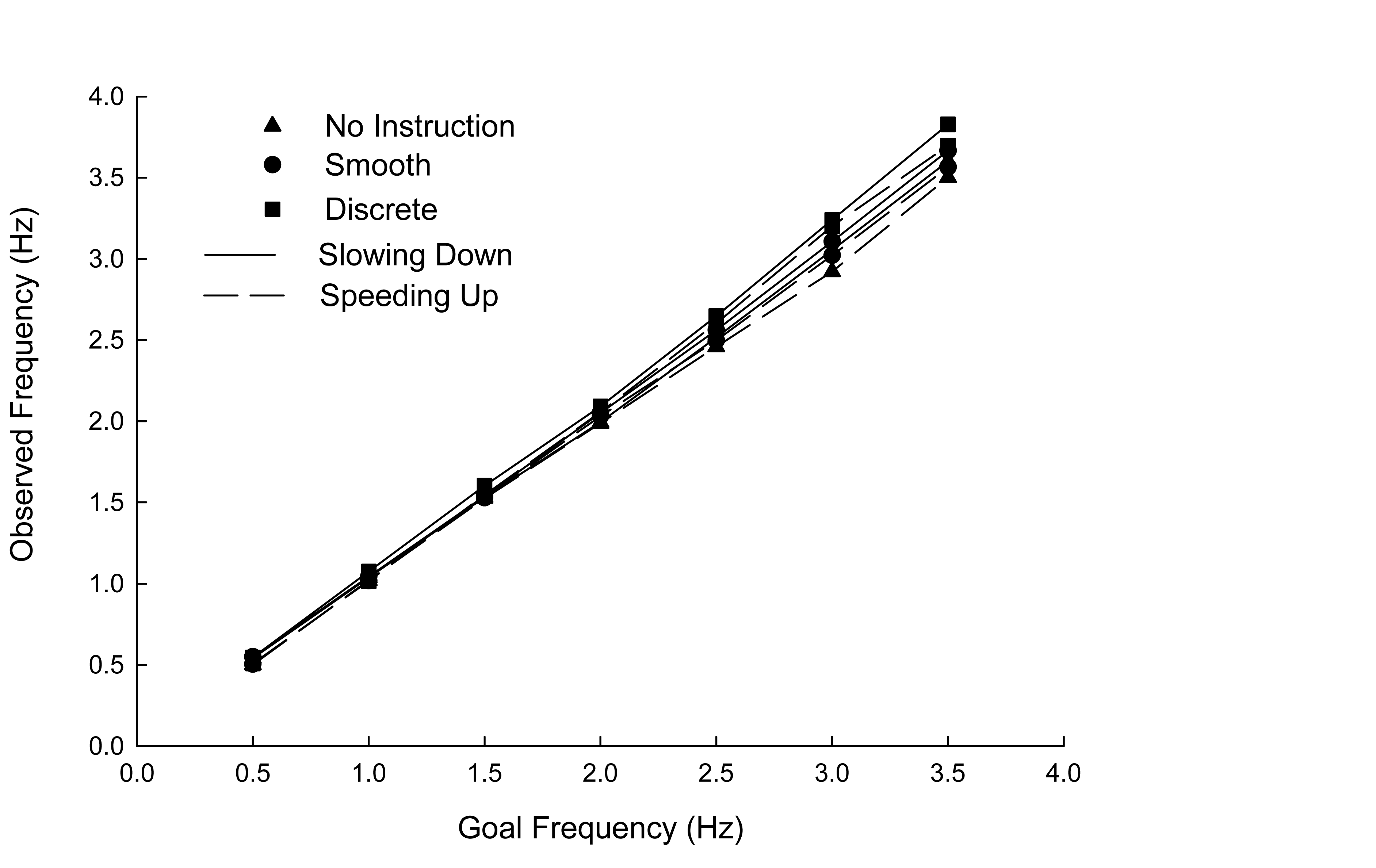

Supplement: Figure S6 — Goal frequency versus observed frequency. Note that in conditions where participants were slowing down, the observed frequency values are plotted in the reverse order of which they were performed. (0.24 MB TIF) [file pcbi.1000061.s006.tif]

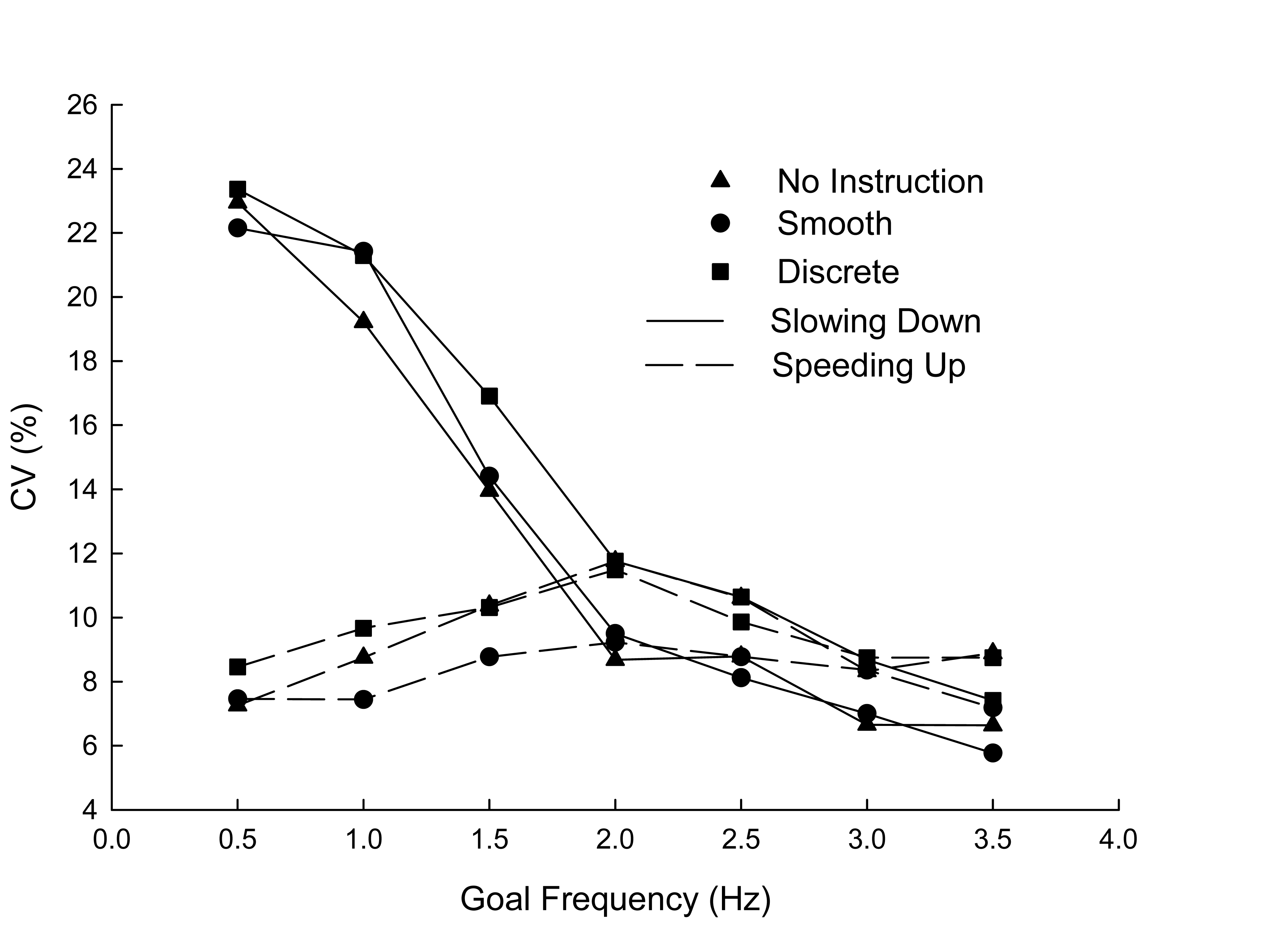

Supplement: Figure S7 — Goal frequency versus coefficient of variation. Note that in conditions where participants were slowing down, the CVs are plotted in the reverse order of which they were performed. (0.27 MB TIF) [file pcbi.1000061.s007.tif]

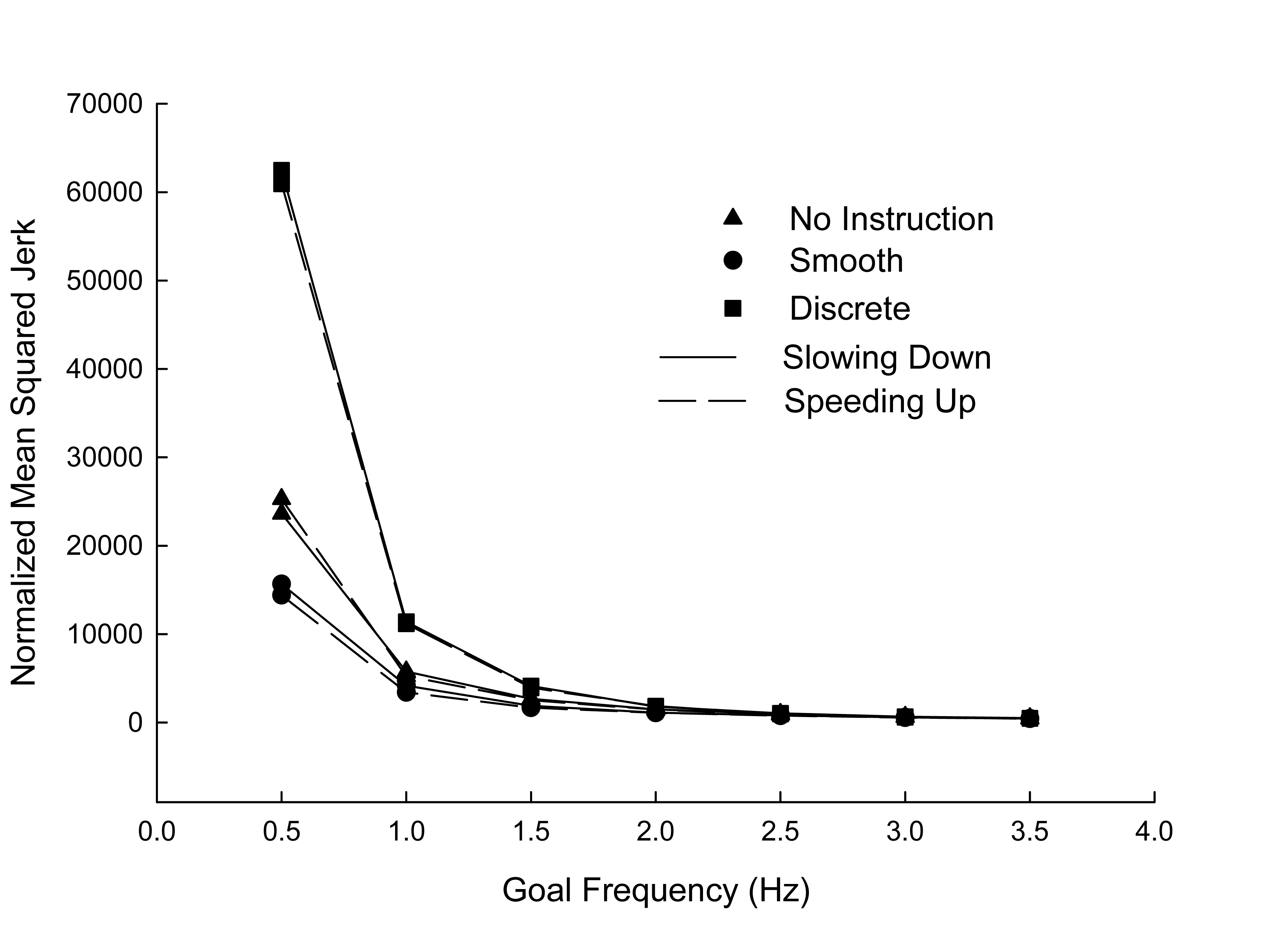

Supplement: Figure S8 — Goal frequency versus normalized mean squared jerk. Note that in conditions where participants were slowing down, the values of jerk are plotted in the reverse order of which they were performed. (0.27 MB TIF) [file pcbi.1000061.s008.tif]

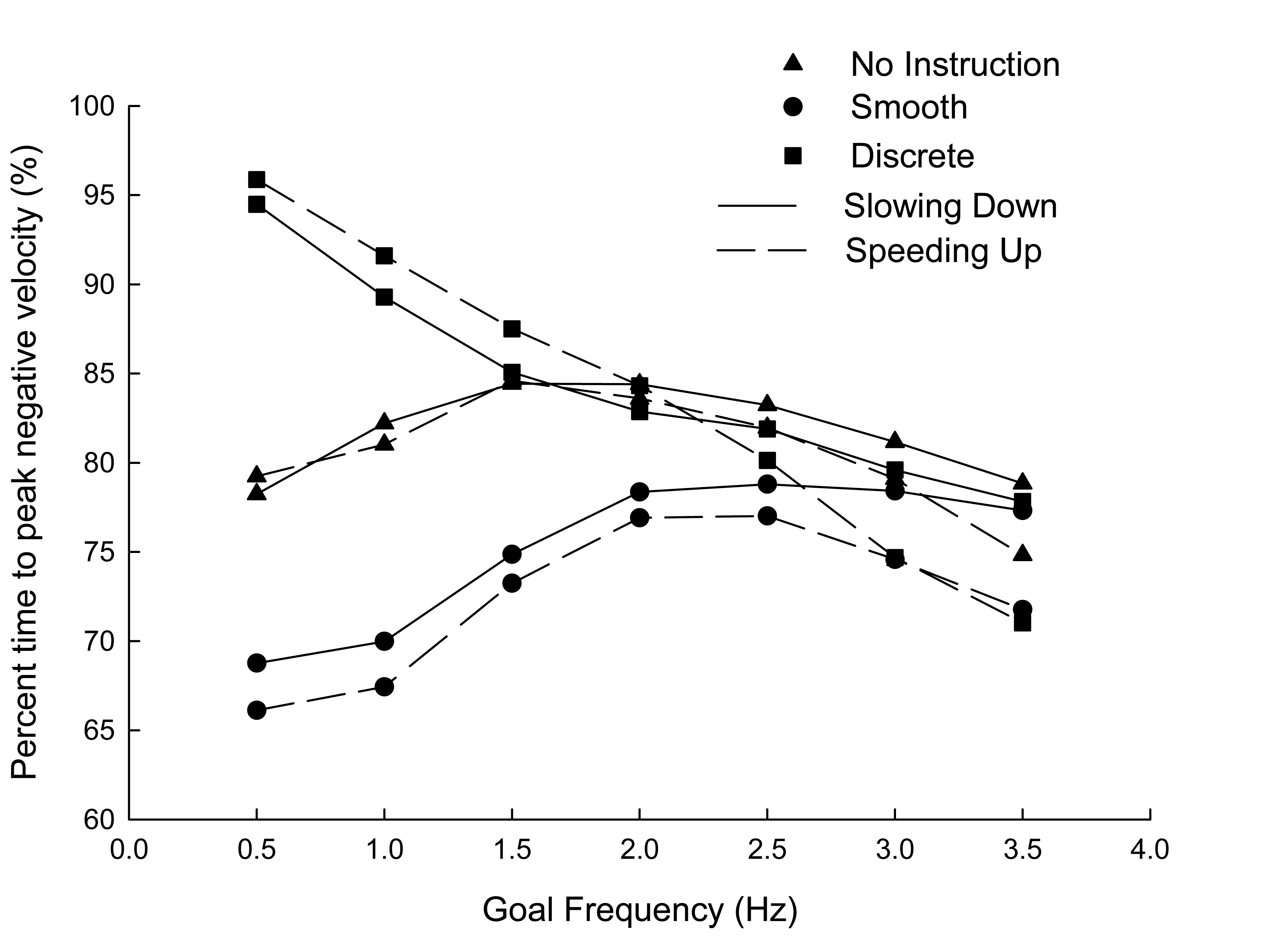

Supplement: Figure S9 — Goal frequency versus percentage of time to peak negative velocity. Note that in conditions where participants were slowing down, the values of percent time to peak negative velocity are plotted in the reverse order of which they were performed. (0.29 MB TIF) [file pcbi.1000061.s009.tif]
