# Supplementary material for: Distinct Timing Mechanisms Produce Discrete and Continuous Movements
Source: PLoS Comput Biol. 2008 Apr 25;4(4):e1000061. doi: 10.1371/journal.pcbi.1000061 (PMC2329590; doi:10.1371/journal.pcbi.1000061)
Supplement: Text S1 — Supporting information. (0.07 MB DOC) [file pcbi.1000061.s010.doc]

**Text S1**

**The excitator model concept**

The generic model equation reads

where and represent the time derivatives of *x* and *y*, respectively, and *τ* and *I* represent a time constant and an (instantaneous) stimulation that is external to the system, respectively. For an appropriate choice of *g_1_*(*x*) and *g_2_*(*x*,*y*), this model belongs to the class of excitable systems [1, 2]. Distinct topologies can be implemented via specific realizations of *g_1_*(*x*) and *g_2_*(*x*,*y*). The realization chosen by Jirsa and Kelso [3] reads *g_1_*(*x*) = *x*^3^/3+ *x*^5^/5 and *g_2_*(*x*,*y*) = *b*∙*y*, which has been obtained from fits to experimental data. The parameters *a* and *b* determine whether the topological structures in phase space are a stable fixed point and a separatrix (the mono-stable regime), two stable fixed points and a separatrix (the bi-stable regime) or a stable limit cycle regime. In terms of movements, these realizations correspond to entire flexion-extension movements, single flexion (or extension) movements, and rhythmic movement, respectively. Here, we concentrate on the realization of the mono-stable regime and limit cycle regime.

**Phase space probability distributions and spectral analysis**

*Method* For the simulated and human data we examined the phase space probability distributions and conducted spectral analysis with the aim to investigate the robustness of the model’s behaviour under different parameter settings, provide a comparison between the model and human data, and to determine whether there was converging evidence for the results reported in the main text. These particular analyses were chosen because they provide insights into the structural features of the concept model (see above) and are relatively robust against different model realization through the functions *g_1_* and *g_2_* (see equation above).

We computed each time series 2-dimensional phase space probability density distribution using a 26 × 26 grid in the (normalized) position-velocity plane (see Methods). Each probability distribution was normalized so that the sum equalled one. Representative probability density distributions can be seen in Figure S1. We next determined each probability distribution’s symmetry ratio by dividing the summed probability of the distribution in the 1:13 × 1:26 grid versus that of the 14:26 × 1:26 grid (i.e., the summed probability of the distribution in the lower and upper half of the position-velocity plane, respectively). In the case of perfect symmetry, this ratio equals one; the larger the deviation from one, the more asymmetrical the probability distribution.

In addition, we computed each time series’ spectral density estimate (*P*), which was subsequently normalized to unit variance. We then determined the amount of spectral power at the fundamental frequency (*ω*), the (first) sub-harmonic (*ω_/2_*), and that of the first super-harmonic (2*ω*). In most instances most spectral power was found at the frequency corresponding to the stimulus frequency in the mono-stable regime. However, the amount of spectral power at 2*ω* relative to the stimulus frequency sometimes exceeded that at the stimulus frequency due to the abruptness of the movement and the ‘sharpness’ of the resulting trajectory. Therefore, for simulation purposes, the spectral power at 2*ω* was determined relative to the stimulus frequency.

*Simulations results* In the mono-stable regime, the symmetry ratio varied primarily as a function of frequency, with values of approximately 15 to 20 at low frequencies (0.25 – 0.5 Hz) that quickly levelled off to values of around one to three at high frequencies (Figure S2). Parameter *a* had a negligible influence only. The symmetry ratio in the limit cycle regime was approximately one (mean ± SD of 1.06 ± 0.06) across all parameters.

The spectral analysis indicated that in the mono-stable regime, the impact of parameters *a* (in particular) and *b* was negligible, albeit that extreme values of parameter *b* slightly affected the spectral distribution. In contrast, the impact of frequency was large. From Figure S3 (left panel) it can be appreciated that the spectral power at the sub-harmonic *ω*/2 is negligible for frequencies below approximately 2.0 Hz (with some dependence on parameter *b*), after which it quickly rises to values larger than one, which reflects a period-doubling (see also Figure 2, main text). (The absence of the period doubling at the extreme end of the parameter *b* range was accompanied by an approximate three-fold amplitude reduction.) In contrast, the spectral power at the harmonic 2*ω* is larger than one, which represents abrupt, ‘sharp’ movement, for frequencies below approximately 2.0 Hz and vanishes for higher frequencies (Figure S3, right panel). The power at the fundamental frequency is maximal at approximately 1.5 to 2.0 Hz and monotonically levels off in both directions (Figure S3, middle panel). For the limit cycle simulations, the spectral power at the fundamental frequency, the sub-harmonic and first super-harmonic varied only marginally as a function of the parameters; that at the fundamental frequency covered approximately all power, while that of the sub-harmonic and first super-harmonic was always low (up to approximately 1% of that at the fundamental frequency).

*Human data results* The across-trial means of all extracted variables were subjected to ANOVA with presentation order (2), instruction (3), and frequency (7) as within participant factors. Main effects were further analyzed via post-hoc tests using Bonferroni adjustments. To anticipate, visual inspection suggested that in the natural condition the participants adopted one of two distinct behaviours in that four (out of eight) participants adopted a ‘discrete’ motor solution at low frequencies. Therefore, all variables were furthermore subjected to repeated measures ANOVA with presentation order (2), instruction (3), and frequency (7) as within participant factors, and motor solution as a between-participant factor.

For the symmetry ratio there was a significant main effect of instruction, *F*(2,14) = 29.390, *p* < .001 (mean ± SE is 4.01±0.51, 5.93±0.43, and 2.72±2.89, for the natural, discrete, and smooth condition, respectively). Post-hoc testing indicated that the three instruction conditions all differed from each other. Furthermore there was a significant main effect of frequency, *F*(6,42) = 58.956, *p* < .001 (mean ± SE is 10.10±1.05, 5.41±0.55, 4.08±0.35, 3.43±0.23, 2.69±0.18, 2.10±0.15, and 1.74±0.13, for 0.5, 1.0, 1.5, 2.0, 2.5, 3.0, and 3.5 Hz, respectively), which indicated that the distribution became more symmetrical as movement frequency increased. Even though the symmetry increase with increasing frequency clearly levelled off, all frequency conditions differed significantly from each other except the 2.0 and 2.5 Hz condition. In addition, we found a significant interaction between instruction and frequency, *F*(12,84) = 34.330,  *p* < .001, which revealed that the frequency effect was pronounced strongest in the discrete condition and weakest in the smooth condition. The ANOVA including motor solutions as a factor revealed (an additional) significant interaction between instruction, frequency, and motor solution, *F*(12,72) = 6.089, *p* < .001, which confirmed the suggestion mentioned above (see Figure S4).

For the sub-harmonic (*ω*/2), there was a significant main effect of instruction, *F*(2,14) = 9.079, *p* = .003 (mean ± SE is 1.00 ×10^-2^ ± 0.10 ×10^-2^, 2.73 ×10^-2^ ± 0.60 ×10^-2^, and 0.95 ×10^-2^ ± 0.10 ×10^-2^, for the natural, discrete, and smooth condition, respectively), which occurred because the discrete condition differed significantly from the other conditions. For the fundamental frequency, there was a main effect of instruction, *F*(2,14) = 81.935, *p* < .001 (mean ± SE is 0.56 ± 0.02, 0.38 ± 0.01, and 0.59 ± 0.01, for the natural, discrete, and smooth condition, respectively), because less spectral power at the fundamental frequency was found in the discrete condition than in the other instruction conditions. Further, a main effect of frequency, *F*(6,42) = 31.408, *p* < .001, (mean ± SE is 0.41±0.02, 0.47±0.02, 0.51±0.01, 0.54±0.01, 0.56±0.01, 0.55±0.02, and 0.55±0.01, for 0.5, 1.0, 1.5, 2.0, 2.5, 3.0, and 3.5 Hz, respectively) was observed, which occurred because the 0.5, 1.0 and 1.5 Hz conditions differed from all others conditions, while the power of the resulting conditions was not significantly different. In addition, the spectral power at the fundamental frequency was approximately constant across frequencies in the smooth condition while it was much lower at low frequencies in the discrete condition but reached a value similar to that in the smooth condition at high frequencies (3.0 and 3.5 Hz) (Instruction × Frequency interaction, *F*(12,84) = 40.174, *p* < .001). A similar but smaller pattern was observed for the natural condition. The ANOVA including motor solution as a between-participant factor revealed a significant interaction between instruction, frequency, and motor solution, *F*(12,72) = 5.690, *p* < .001, as shown in Figure S5 (left panel). This interaction is due to the observation that the spectral power at the fundamental frequency for the participants who had adopted the ‘smooth’ solution in the natural condition was approximately constant across all frequencies while for those that had adopted the ‘discrete’ solution it increased with increasing frequency similar (but weaker) than in the discrete condition.

Finally, for the first super-harmonic (2*ω*) a main effect instruction occurred, *F*(2,14) = 66.134, *p* < .001 (mean ± SE is 0.07 ± 0.02, 0.25 ± 0.02, and 0.01 ± 0.00, for the natural, discrete, and smooth condition, respectively). All three instruction conditions differed significantly from each other. In addition, there was an effect for frequency, *F*(6,42) = 82.534, *p* < .001 (mean ± SE is 0.28±0.03, 0.21±0.02, 0.14±0.01, 0.08±0.01, 0.04±0.01, 0.02±0.00, and 0.01±0.00, for 0.5, 1.0, 1.5, 2.0, 2.5, 3.0, and 3.5 Hz, respectively), and revealed that that the 2.5 Hz and 3.0 Hz conditions did not differ significantly from the 3.5 Hz condition while all other conditions were significantly different. There also was a significant interaction between instruction and frequency, *F*(12,84) = 38.023, *p* < .001. This interaction can be appreciated by considering the three-way interaction between instruction, frequency, and motor solution, *F*(12,72) = 8.310, *p* < .001 (see Figure S5 right panel). This interaction revealed the same, albeit reversed pattern, as that for the fundamental frequency. Finally, there was a significant Instruction × Motor solution interaction, *F*(2,12) = 5.261, *p* = .023, as well as a Frequency × Motor solution interaction, *F*(6,6) = 4.592, *p* = .001. (The latter two interactions can be readily understood from the three-way interaction and will therefore not be discussed).

*Discussion* The analysis of symmetry ratio of the simulations indicated that the model behaviour was robust across a wide range of parameters, and revealed a change as a function of frequency for the mono-stable regime (only). The visually apparent match between the mono-stable simulations and the discrete condition (human data) on the one hand and the limit cycle simulations and the human performances in the smooth condition on the other hand was confirmed by the analysis. For the natural condition, the dependence of the symmetry ratio on frequency depended on the motor solution adopted; the symmetry of the probability distributions either decreased with increasing frequency (for the ‘discrete’ solution) or remained more or less constant across frequencies (for the ‘smooth’ solution). These results strongly suggest that the participants’ behaviour in the discrete condition (and for some in the natural condition) was governed by limit cycle dynamics. Because the symmetry ratio of the mono-stable simulations and for the human data levelled off to values observed for the limit cycle regime, this analysis cannot provide any insight into the control underlying human behaviour at high frequencies.

In contrast to the model data in the mono-stable regime, the spectral analysis of the human data never revealed a period doubling (relative to the metronome frequency) with increasing frequency. This result strongly suggests that the participants did not adopt a fixed point dynamics at high frequencies. In contrast, the trade-off in spectral content between the fundamental frequency and the first harmonic from low to intermediate frequencies (around 2.0 Hz) was similarly present in the mono-stable simulations as in the human data, albeit to a lesser degree. In that regard, let us note that it seems reasonable to suggest that the phase flows are implemented on the neural level conveying efferent signals to the peripheral movement dynamics. While the relation between these two levels of organization appears to be linear [4-7], some filtering (or masking) of the phase flows seems inevitable. Regardless, qualitative phenomena of the respective phase flows should be preserved. Further, the strong and apparently linear correlation between specific neural activity and motor behavior suggests that any filtering effect will only mask the neural dynamics quantitatively in small parameter regimes, but preserve qualitative features, effectively introducing merely an uncertainty where the transition from one dynamical regime to the other occurs.

Taken together, the analysis of the model’s behaviour provide converging evidence (to the analysis in the main text) that whenever the human participants adopted a fixed point dynamics at slow movement paces (i.e., in the discrete condition and for some participants in the natural condition) they invariantly switched to a limit cycle dynamics at frequencies larger than approximately 2.0 Hz.

**Descriptive Movement Behavior**

In order to provide a complete record of the behavior of our research participants, we present descriptive kinematic data. These results show that our research participants performed the required tasks. The behavioral data were examined in terms of variables commonly found in the literature, namely, and cycle duration and its coefficient of variation (CV; [8, 9]), normalized mean squared jerk [10-12] and percent time to peak negative velocity [12, 13] were calculated. Velocity was defined negative for finger movements in the flexion direction. Cycle onset was defined as the moment at which the (negative) velocity exceeded 3% of the maximal (negative velocity). The CV (i.e., mean divided by the stand deviation) is presented as a percentage (i.e., it is multiplied by100). We computed jerk (the derivative of acceleration) and subsequently normalized the jerk to the period and movement amplitude [11]. Finally, the percentage to peak velocity was computed over the flexion movement as the task was to time full flexion with the occurrence of the auditory tone. For all variables (except CV), the values were calculated on a cycle by cycle basis, averaged within a duration (seven different movement rates within a trial), and then averaged across trials and participants for each unique condition. These average values were subjected to ANOVAs with (presentation) order (2), instruction (3), and frequency (7) as within participant factors.

Observed movement frequency was approximately that of the prescribed metronome period in all conditions at each frequency step condition. There was a significant effect of frequency, *F*(6,42) = 10442, *p* < .0001 ,as expected. There was also a significant effect of order *F*(1,7) = 48.78, *p* = .0002; trials that began at 0.5 Hz and ended at 3.5 Hz had an overall slower cycle duration (731 ms) than trials that began at 3.5 Hz and ended at 0.5 Hz (699 ms). There was also a significant order by frequency interaction (see Figure S6), *F*(6,42) = 28.08, *p* <.0001, which indicated a difference between the slowing down and speeding up (the order variable) condition at the slowest movement rate (0.5 Hz) but not at the faster movement rates (3.5 to 2.5 Hz).

For coefficient of variation (CV) there was a significant main effect of order, *F*(1,7) = 145.34, *p* < .0001, and frequency, *F*(6,42) = 117.87, *p* < .0001. There was no significant effect of instruction for the CV. There was a significant interaction between order and frequency (see Figure S7; *F*(6,42) = 71.26,  *p*< .0001). Tasks performed at slower movement rates had larger CVs (23% at 0.5 Hz) than those performed at faster movement rates (7% at 3.5 Hz) only for the slowing down condition. For the speeding up condition, the CV stayed between 6% and 12% for all durations (8% at .5 Hz (2000 ms period) and 8% at 3.5 Hz (286 ms period)).

For the normalized mean squared jerk, there was a significant main effect of instruction condition, *F*(2,14) = 86.73, *p* < .0001, and frequency, *F*(6,42) = 336.70, *p* < .0001. The discrete instruction condition produced a greater value of normalized mean squared jerk (11563) while moving smoothly induced the smallest values of NMSJ (3351). In the no instruction conditions, participants had values of NMSJ similar to when given the smooth instruction (5152). The value of NMSJ increased as movement frequency increased, and this occurred to a larger extent for the discrete conditions than for the smooth and no instruction conditions. This observation was supported by a significant instruction by frequency interaction (see Figure S8; *F* = (12,84) = 82.44, *p* < .0001). There were no differences in values of NMSJ for the speeding up and slowing down orders.

For percent time to peak negative velocity there were significant main effects for all conditions. Participants achieved peak (negative) velocity at about 81% of the movement cycle for the no instruction condition, at about 84% of the movement cycle for the discrete instruction condition and about 74% of the movement cycle for the smooth instruction condition (see Figure S9; *F*(2,14) = 27.51, *p* < .0001). Peak (negative ) velocity was achieved at about 80% of the movement cycle on trials that slowed down and at about 79% of the movement cycle on trials that sped up, *F*(1,7) = 7.89, *p* = .03. There were significant interactions between instruction and frequency, *F*(12,84) = 22.71, *p* <.0001, order and frequency, *F*(6,42) = 9.92, *p* <.0001, and instruction by order by frequency, *F*(12,84) = 2.45, *p* = .009. In general, time to peak velocity was between 75% and 80% for movement frequencies between 3.5 and 2.0 Hz (at 3.5 Hz; 77% no instruction, 75% smooth instruction, 74% discrete instruction). For 1.5, 1.0 and 0.5 Hz, time to peak negative velocity changed as a function of instruction with large separation between instruction conditions for movement at .5 Hz (79% no instruction, 67% smooth instruction, 95% discrete instruction).

**References Supporting Information**

1. FitzHugh R (1961) Impulses and physiological states in theoretical models of nerve membrane. J Biophys 1: 445-466.

2. Murray JD (1993) Mathematical Biology. New York: Springer.

3. Jirsa VK, Kelso JAS (2005) The Excitator as a minimal model for the coordination dynamics of discrete and rhythmic movement generation. J Mot Behav 37: 35-51.

4. Kelso JAS, Fuchs A, Lancaster R, Holroyd T, Cheyne D *et al*. (1998) Dynamic cortical activity in the human brain reveals motor equivalence. Nature 392: 814-818.

5. Jirsa VK, Fuchs A, Kelso JAS (1998) Connecting cortical and behavioral dynamics: bimanual coordination. Neural Comp 10: 2019-2045.

6. Beek PJ, Peper CE, Daffertshofer A (2002) Modeling rhythmic interlimb coordination: beyond the Haken-Kelso-Bunz model. Brain & Cognition 48: 149-165.

7. Schwartz AB, Moran DW, Reina AG (2004) Differential representation of perception and action in the frontal cortex. Science 303: 380-383.

8. Spencer RMC, Zelaznik HN, Diedrichsen J, Ivry RB (2003) Disrupted timing of discontinuous but not continuous movements by cerebellar lesions. Science 300: 1437-1439.

9. Zelaznik HN, Spencer RMC, Ivry RB, Baria A, Bloom M *et al*. (2005) Timing variability in circle drawing and tapping: Probing the relationship between event and emergent timing. J Mot Behav 37: 395-403.

10. Van Mourik AM, Beek PJ (2004) Discrete and cyclical movements: Unified dynamics or separate control? Acta Psych 117: 121-138.

11. Teulings H-L, Contreras-Vidal JL, Stelmach GE, Adler CH (1997) Parkinsonism reduces coordination of fingers, wrist, and arm in fine motor control. Exp Neurol 146:159-170.

12. Balasubramaniam R, Wing AM, Daffertshofer A (2004) Keeping with the beat: movement trajectories contribute to movement timing. Exp Brain Res 159: 129-134.

13. Gentili R, Cahouet V, Papaxanthis C (2007) Motor planning of arm movements is direction-dependent in the gravity field. Neurosci 145: 20-32.
